# Type II Collagen and Gelatin from Silvertip Shark (*Carcharhinus albimarginatus*) Cartilage: Isolation, Purification, Physicochemical and Antioxidant Properties

**DOI:** 10.3390/md12073852

**Published:** 2014-06-27

**Authors:** Elango Jeevithan, Bin Bao, Yongshi Bu, Yu Zhou, Qingbo Zhao, Wenhui Wu

**Affiliations:** Department of Marine Pharmacology, College of Food Science and Technology, Shanghai Ocean University, Shanghai 201306, China; E-Mails: srijeevithan@gmail.com (E.J.); bruce.bu@unilever.com (Y.B.); yzhou0724@163.com (Y.Z.); bobozhao123@163.com (Q.Z.)

**Keywords:** shark cartilage, type II collagens, denaturation temperature, FTIR, SEM, antioxidant activity

## Abstract

Type II acid soluble collagen (CIIA), pepsin soluble collagen (CIIP) and type II gelatin (GII) were isolated from silvertip shark (*Carcharhinus albimarginatus*) cartilage and examined for their physicochemical and antioxidant properties. GII had a higher hydroxyproline content (173 mg/g) than the collagens and cartilage. CIIA, CIIP and GII were composed of two identical α_1_ and β chains and were characterized as type II. Amino acid analysis of CIIA, CIIP and GII indicated imino acid contents of 150, 156 and 153 amino acid residues per 1000 residues, respectively. Differing Fourier transform infrared (FTIR) spectra of CIIA, CIIP and GII were observed, which suggested that the isolation process affected the secondary structure and molecular order of collagen, particularly the triple-helical structure. The denaturation temperature of GII (32.5 °C) was higher than that of CIIA and CIIP. The antioxidant activity against 1,1-diphenyl-2-picrylhydrazyl radicals and the reducing power of CIIP was greater than that of CIIA and GII. SEM microstructure of the collagens depicted a porous, fibrillary and multi-layered structure. Accordingly, the physicochemical and antioxidant properties of type II collagens (CIIA, CIIP) and GII isolated from shark cartilage were found to be suitable for biomedical applications.

## 1. Introduction

Type II collagen is a principal component of the extracellular matrix of articular cartilage and constitutes 90%–95% of the total protein content in the cartilage [1]. In the current commercial market, most of the type II collagen is isolated from terrestrial mammalian cartilage due to its high biocompatibility, and it is widely used in the pharmaceutical, food, healthcare, and cosmetic industries [2]. However, the incidences of diseases such as bovine spongiform encephalopathy (BSE) and foot and mouth disease (FMD) have raised concerns about its safety. Alternatively, collagen from fish processing waste may be a suitable substitute. Production of fish collagen and gelatin not only adds significant value to the fish processing sector but also to other pharmacological industries.

Gelatin, a denatured form of collagen, has been extensively studied [3,4]; however, studies related to fish collagen are limited to isolation and characterization [2,5,6]. The functional properties of collagen and gelatin are highly influenced by their molecular structure, amino acid composition and internal linkages, which are affected by the processing conditions.

Recently, several researchers have focused on the oral tolerance of type II collagen to treat autoimmune diseases [1,7]. Oral tolerance is an immunological mechanism by which external agents that enter the body through the digestive system are recognized and ignored by the immune system. The effects of oral administration of type II collagen obtained from bovine, chicken and sheep sources have been evaluated for the treatment of rheumatoid arthritis (RA) [8,9,10].

The antioxidant activity of collagen is an essential property for the oral tolerance mechanism in autoimmune diseases [11]. Recently, an interest in natural antioxidants has increased because they are widely distributed and safer than synthetic antioxidants. Few studies have been conducted on the antioxidant activity of collagen and gelatin isolated from marine animals, and include sea cucumber skin [12,13], jellyfish skin [14]; squid skin [15] and tuna skin and bone [16]. Recently, Merly and Smith [17] studied the immunomodulatory properties of type II collagen from commercially available shark cartilage capsules. To our knowledge, the physicochemical and antioxidant activities of silvertip shark (*Carcharhinus albimarginatus*) cartilage type II collagen and gelatin have not been examined. Therefore, the present study investigated the physicochemical and antioxidant properties of acid- and pepsin-soluble type II collagens (ASC, PSC) and type II gelatin from silvertip shark cartilage and their potential for further applications.

## 2. Results and Discussion

### 2.1. Biochemical Composition of Shark Cartilage

The cartilage ash content was high (25.41%) compared with the protein content (8.95%) (data not shown). This high level of ash was also reported in the cartilage of Amur sturgeon and Nile perch (26.7% and 39.1%, respectively) [18,19]. The generally high ash content of shark cartilage is attributed to its high mineral content [3]. A protein content of 19.2% was reported for another shark species (*Isurus oxyrinchus*) cartilage [20] and was higher than our finding. The moisture and fat content of the silvertip shark cartilage was 63.56% and 1.69%, respectively. Cho *et al.* [20] reported that the fat content of *I. oxyrinchus* shark cartilage was 1.4%, which was lower than in the present report. The moisture, protein, ash and fat contents of cartilage of several species of shark are between 66.84%–78.3%, 14.01%–19.20%, 1.10%–12.09% and 0.21%–1.40%, respectively [20,21]. Fish cartilage generally contains a lower protein content than in skin due to higher ash and fat contents [3].

The hydroxyproline content of shark cartilage was 30.28 mg/g (data not shown), which was lower than that found in CIIA, CIIP and GII. A lower hydroxyproline content of 11.37–13.44 mg/g for shark cartilage was reported by Kittiphattanabawon *et al.* [21]. Variations in the hydroxyproline content among fish species occur because of the differences in their body temperature and seasonal variations. The hydroxyproline content of CIIA, CIIP and GII were 92.7, 113 and 173 mg/g, respectively. Kittiphattanabawon *et al.* [21] reported that hydroxyproline content of PSC and ASC from brownbanded bamboo shark (*Chiloscyllium punctatum*) cartilage was between 103.71 and 104.49 mg/g, which is similar to our findings. Accordingly, silvertip shark cartilage may be a better resource for the isolation of collagen.

### 2.2. Protein Pattern

**Figure 1:**
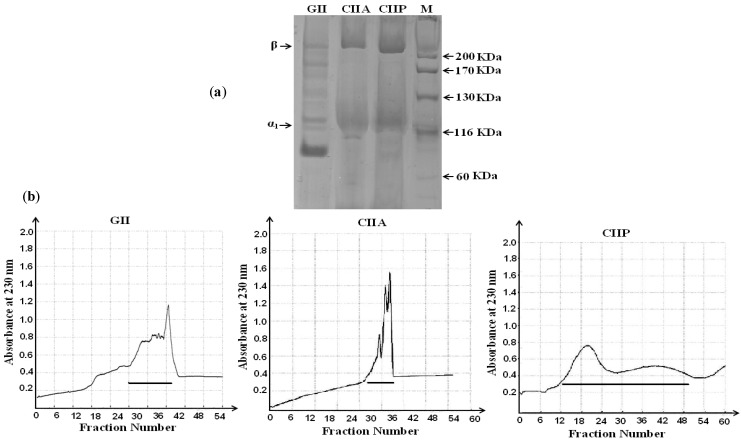
(**a**) Electrophoretic pattern of type II collagens and type II gelatin isolated form shark cartilage; (**b**) Purification of type II collagen and type II gelatin by gel filtration chromatography. The horizontal lines in the chromatograms represent pooled fractions that were analyzed by SDS-PAGE. GII: type II gelatin, CIIA: type II acid soluble collagen, CIIP: type II pepsin soluble collagen.

The degree of purification by gel filtration chromatography and the protein pattern of CIIA, CIIP and GII were evaluated by SDS-PAGE. The purified CIIA and CIIP showed two distinct bands, α_1_ and β, with molecular weights of approximately 120 and 210 kDa, respectively (Figure 1a).

The band intensity of the α_1_-chain was not greater than the β chain in CIIA and CIIP. This may be due to the formation of a β dimer resulting from the inter- and intramolecular cross-linking of the α component in the collagen structure. A similar protein pattern for type II collagen isolated from the cartilage of sharks and chicks has been reported in several studies [21,22,23]. When compared with CIIA and CIIP, the mobility of the GII α_1_-chain was lower (approximately 100 kDa) and the band intensity was higher, which may indicate a conversion of the β dimer into the α component in the heat extraction process. In addition, smaller MW fractions were observed in GII in between the α component and β dimer, which was attributed to the denaturation of collagen during the isolation process. Therefore, type II collagen isolated from shark cartilage is pure and is composed of two chains, such as α_1_ and β, and the secondary structure of collagen was altered by heat extraction. 

### 2.3. Peptide Mapping

**Figure 2:**
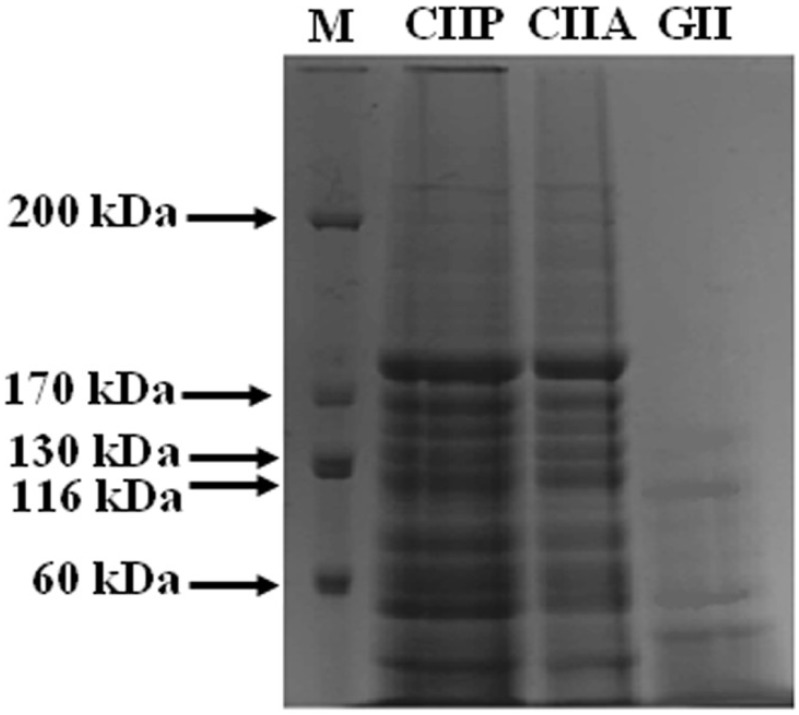
Peptide map of collagen and gelatin isolated form shark cartilage. CIIP: type II pepsin soluble collagen, CIIA: type II acid soluble collagen, GII: type II gelatin, M: Marker.

Shark cartilage type II collagens (CIIA and CIIP) digested by the V8 protease from *Staphylococcus aureus* V8 (EC 3.4.21.19, Sigma–Aldrich, Shanghai, China) exhibited a similar pattern. After hydrolysis, the α chain and β component of the collagens were degraded into small molecular weight peptides ranging from 180 to 40 kDa (Figure 2). This was in accordance with the peptide pattern for brownbanded bamboo shark cartilage collagen reported by Kittiphattanabawon *et al.* [21]. Peptide mapping revealed that CIIA and CIIP had similar secondary structures. The V8 protease exhibits a high degree of specificity for glutamic acid and aspartic acid residues in proteins [24]. The contents of glutamic acid and aspartic acid residues were higher in CIIP (76.20 and 45.76 residues per 1000 residues, respectively) than CIIA (71.57 and 42.91 residues per 1000 residues, respectively). Therefore, CIIP was more susceptible to hydrolysis by the V8 protease than CIIA, although, a similar pattern of peptide fragments were observed. This result was in accordance with the peptide mapping of ASC and PSC extracted from the skin of the brownstripe red snapper [25].

GII exhibited a different peptide pattern than CIIA and CIIP. The α_1_-chain, as well as high MW cross-linked β components, of GII were completely degraded by the V8 protease and thus more small peptides were observed. This suggests that the α_1_-chain and β components of GII were more susceptible to the V8 protease than CIIA and CIIP. Although GII had lower glutamic acid and aspartic acid contents than the collagens, the degree of hydrolysis was higher in GII. This may be due to differences in the accessibility of susceptible bonds to the proteinase and to differences in the secondary structure between the collagens and gelatin, which were observed in the FTIR spectra. Hence, the peptide pattern of type II collagens (PSC, ASC) and type II gelatin from shark cartilage may be influenced by internal cross-linking, secondary structure and the composition of amino acids.

### 2.4. Maximum Absorption

The maximum absorptions of CIIA, CIIP and GII were 238.6, 237.7 and 237.6 nm, respectively (data not shown). The maximum absorption reported for eel skin collagen was similar to our findings [26]. In the present study, shark type II collagens and type II gelatin did not exhibit an absorption peak at 280 nm. This was due to a low tyrosine content (2–8 residues per 1000 residues), which absorbs UV light at 280 nm [27]. Kittiphattanabawon *et al.* [21] stated that no absorption peak of collagen at 280 nm indicates the purity of the collagen and efficacy of non-collagenous protein removal. This indicates adequate efficiency of the collagen and gelatin isolation processes used in this study.

### 2.5. Amino Acid Composition

CIIA, CIIP and GII extracted from shark cartilage had similar amino acid profiles (Table 1). In general, CIIA, CIIP and GII had high glycine contents (326, 319 and 353 residues per 1000 residues, respectively), which was the primary amino acid followed by alanine and proline. This result was in accordance with other fish collagen studies [18,20,25]. Every third amino acid residue was glycine in the α chain of collagen, except in the first 14 amino acid residues of the *N*-terminus and the first 10 amino acid residues of the *C*-terminus [28]. Higher contents of glycine, alanine and hydroxyproline were observed in GII compared with CIIA and CIIP. Type II collagens and type II gelatin had low contents of Met, His, Cys and Tyr, which is similar to other fish collagens [2,29,30]. The difference in the amino acid composition between type II collagens and type II gelatin was due to the different isolation processes.

Compared with type II collagens and type II gelatin, shark cartilage contained a lower content of glycine, alanine and proline (310, 122 and 93 residues per 1000 residues, respectively), but a higher amount of glutamic acid, aspartic acid, threonine, tyrosine and cysteine. The removal of non-collagenous protein during the isolation process likely resulted in the changes in the amino acid composition between the raw material and collagens. Imino acid contents of shark cartilage, type II collagens and type II gelatin were from 15.6% to 14.3%, which was relatively lower than that of ASC (21%) and PSC (22%) isolated from brownstripe red snapper skin [25]. CIIA had a lower imino acid content than CIIP and GII. The higher imino acid content of PSC compared with ASC was due to the removal of telopeptides by pepsin digestion [25].

**Table 1:** Amino acid compositions of ASC, PSC and gelatin from shark cartilage (residues/1000 residues). CIIP: type II pepsin soluble collagen, CIIA: type II acid soluble collagen, GII: type II gelatin.

Amino Acids	Shark Cartilage	CIIA	CIIP	GII
Hyp	49.96	47.50	49.25	51.28
Asp	51.84	42.91	45.76	39.84
Thr	27.26	23.54	25.57	21.85
Ser	35.62	36.37	38.27	32.62
Glu	86.45	71.57	76.20	71.70
Pro	93.61	103.30	106.78	102.31
Gly	310.47	326.90	319.69	353.12
Ala	122.87	133.92	132.63	140.45
Cys	5.88	3.93	4.26	3.74
Val	25.63	25.14	25.43	22.65
Met	13.69	10.68	13.54	12.34
Ile	18.55	21.19	21.81	16.94
Leu	40.16	30.01	29.95	25.16
Tyr	15.13	8.75	7.19	2.73
Phe	19.51	18.95	14.97	14.66
Lys	22.34	30.93	29.37	27.35
His	9.98	9.33	9.38	8.15
Arg	50.96	55.05	49.87	53.14
Total	1000	1000	1000	1000
Imino acid	143.58	150.81	156.03	153.59

### 2.6. Relative Viscosity

**Figure 3:**
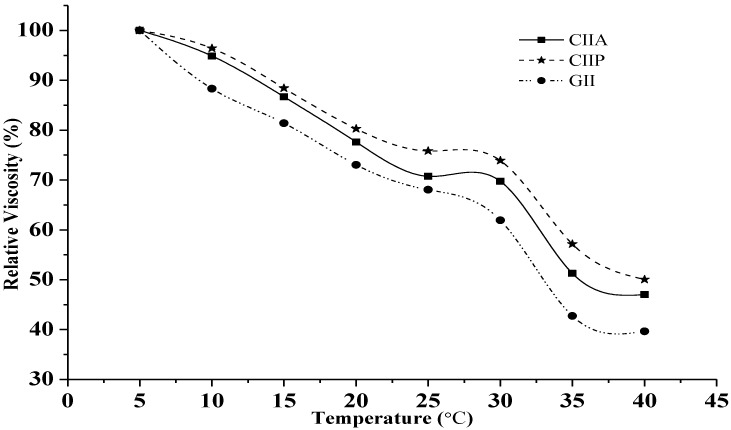
Relative viscosity of type II collagens and type II gelatin. The viscosity obtained at 5 °C was considered as 100%. CIIP: type II pepsin soluble collagen, CIIA: type II acid soluble collagen, GII: type II gelatin.

Relative viscosity is a physicochemical property of collagen. The viscosity of collagen and gelatin decreased continuously with increasing temperature, and this trend was similar for both collagens and gelatin. A sharp decline in the relative viscosity of collagen and gelatin was found above 30 °C (Figure 3). The viscosity observed in this study was similar to that reported for other fish collagens [2,26,31]. The increasing temperature breaks hydrogen bonds between adjacent polypeptide chains of the collagen molecules and transforms intact trimers into individual chains or dimers, and, ultimately, causes the denaturation of the collagen structure. As a result, the helical structure of collagen converts into a random coil with a reduced viscosity. Hence, the viscosity is primarily influenced by the secondary structure of type II collagens and type II gelatin. Viscosity of GII was lower than type II collagens, and this is due to the lower molecular weight and proportion of β chains, as well as to the loss of the triple helical structure of gelatin during the denaturation process [2].

### 2.7. Effect of pH and NaCl Concentration on Solubility

**Figure 4:**
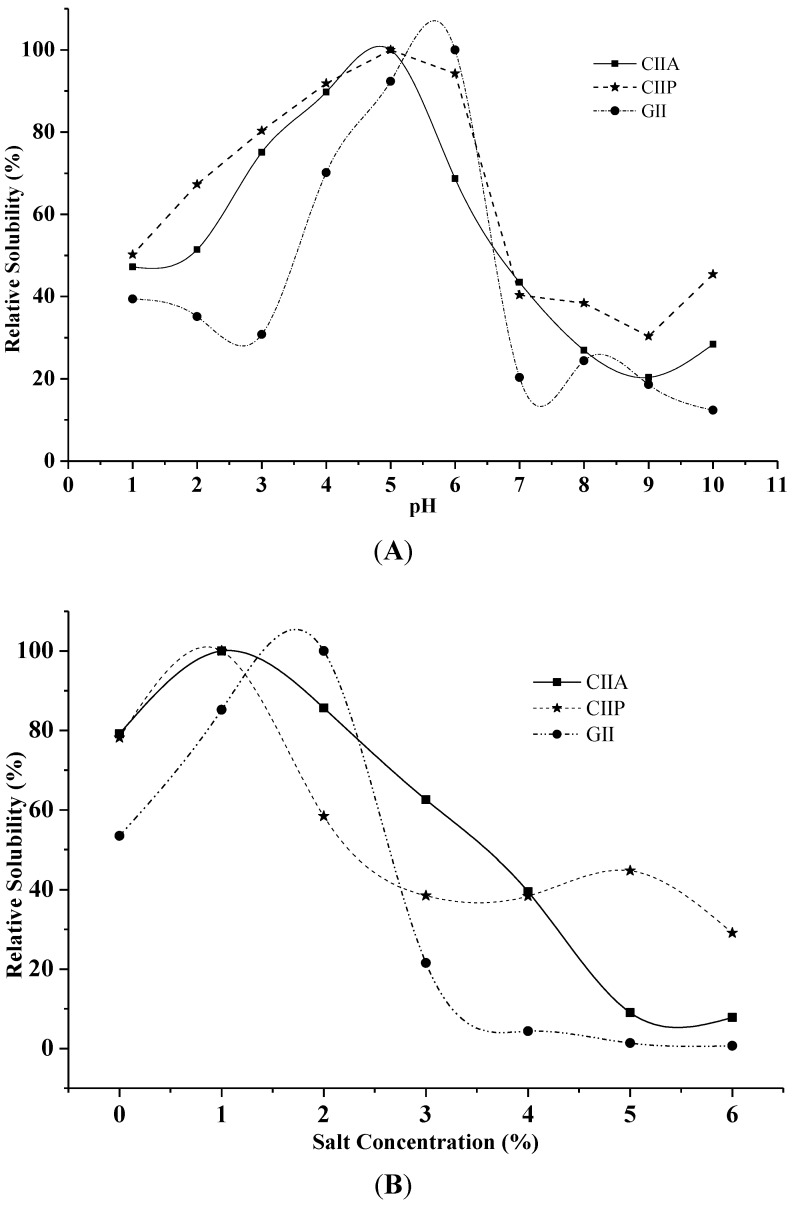
Effect of pH (**A**) and salt concentration (**B**) on the solubility of collagens and gelatin. The highest solubility of collagens and gelatin was considered as 100% solubility. CIIP: type II pepsin soluble collagen, CIIA: type II acid soluble collagen, GII: type II gelatin.

The solubility of CIIA, CIIP and GII increased with increasing pH up to 5 and 6, and above this pH, the solubility decreased (Figure 4A).

Low solubilization of type II collagens and type II gelatin was observed in an alkaline pH from 8 to 9. Bae *et al.* [29] reported that decreased solubility between pH 7 and 9 was due to protein precipitation and the increased relative viscosity of collagen. Moreover, Kittiphattanabawon *et al.* [28] suggested that the variation in solubility at varying pH is attributed to the differences in the molecular conformations of collagen.

Maximum solubility was observed at the salt concentration of 1% for type II collagens and 2% for type II gelatin (Figure 4B). A drastic decrease in solubility was observed between 3% and 4% salt concentration for CIIA and GII and between 1% and 2% for CIIP. The maximum solubility of ASC and PSC from eel skin was reported at 3%–4% salt concentration [26], which is slightly higher than our findings. Jongjareonrak *et al.* [25] explained that the addition of salt decreased the solubility of collagens by increasing the ionic strength and enhancing hydrophobic interactions between protein chains, which leads to protein precipitation. In the present study, CIIP was more soluble than CIIA at 1% salt concentration, which was due to the partial hydrolysis of high molecular weight cross-linked CIIP by pepsin. Variations in the solubility may also be due to differing hydrophobic amino acid contents and the isoelectric point of collagen and gelatin [25].

### 2.8. Thermal Stability

**Figure 5:**
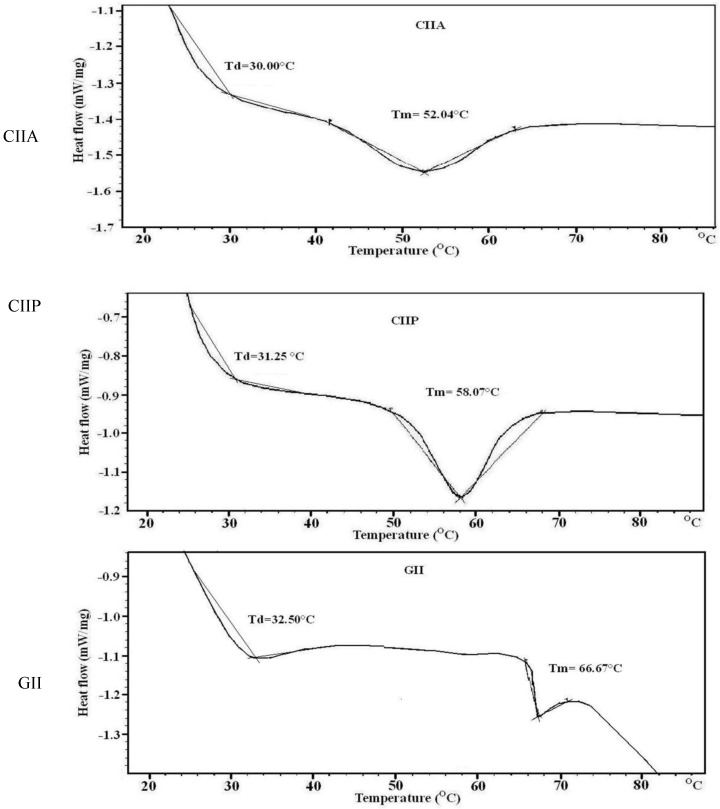
DSC thermograms of type II collagens and type II gelatin. CIIP: type II pepsin soluble collagen, CIIA: type II acid soluble collagen, GII: type II gelatin.

The denaturation (Td) and melting temperatures (Tm) of type II collagens and type II gelatin was determined by DSC. Thermal denaturation denotes unfolding of the triple helix to a random coil and leads to a loss of the unique characteristics of collagens. Denaturation and melting temperature of type II collagens and gelatin are shown in Table 2 and Figure 5. Type II collagens and type II gelatin had two endothermic peaks: the first peak occurred with the thermal denaturation of collagen and the second peak with the breakage of peptide chains and the destruction of the material (melting temperature). According to the DSC spectra, the melting temperature of CIIA, CIIP and GII was observed at 52.04, 58.07 and 66.67 °C, respectively. Wang *et al.* [31] suggested that the difference in thermal stability of ASC and PSC from Amur sturgeon skin may be due to the level of hydration and the number and nature of covalent cross-linkages.

**Table 2:** Antioxidant and thermal transition temperature of type II collagens and type II gelatin. CIIP; type II pepsin soluble collagen, CIIA: type II acid soluble collagen, GII: type II gelatin.

Sample	DPPH Radical Scavenging (%)	Reducing Power (Absorbance at 700 nm)	DSC	
			Denaturation Temp (°C)	Melting Temp (°C)
CIIA	20.08	0.22	30.00	52.04
CIIP	24.77	0.24	31.25	58.07
GII	16.56	0.20	32.50	66.67
BHT	65.72	0.28	-	-

The denaturation temperature (Td) of GII (32.50 °C) was higher than that of CIIP (31.25 °C) and CIIA (30.00 °C). The Td of other fish and calf collagen ranged from 19.4 to 40.8 °C [19,23,26,30]. The denaturation temperature may be affected by the degree of hydroxylation of the Pro and the Gly-Pro-Hyp sequence in collagen and gelatin [24]. The pyrrolidine rings of imino acids restrict the polypeptide chain conformation and strengthen the triple helix. Imino acid content is primarily responsible for the thermal stability and the triple stranded helix formation [32]. However, GII had a lower imino acid content than CIIP and a higher denaturation temperature. Similarly, Li *et al.* [6] reported that porcine collagen had higher thermal stability than Amur sturgeon collagen but a lower imino acid content; these authors proposed that the molecular conformation, amino acid sequence and stable covalent intra- and intermolecular cross-linkages may also influenced the thermal stability of collagen. In the present study, we have determined that the changes in the denaturation temperature are due to the different molecular conformations of type II collagens and type II gelatin.

Bae *et al.* [29] stated that the Td of fish collagen above 33 °C was considered as high heat resistance. In the present study, the Td of shark type II collagens and type II gelatin indicated higher stability and heat resistance than other reported fish collagens. Although, fish collagen has several important biochemical properties, the lower denaturation temperature of fish collagen than mammalian collagen is a major drawback in practical applications. However, the Td of type II collagens and type II gelatin from silvertip shark is similar to mammalian collagen and therefore may be a suitable alternative source of mammalian collagen.

### 2.9. FTIR Spectra

**Figure 6:**
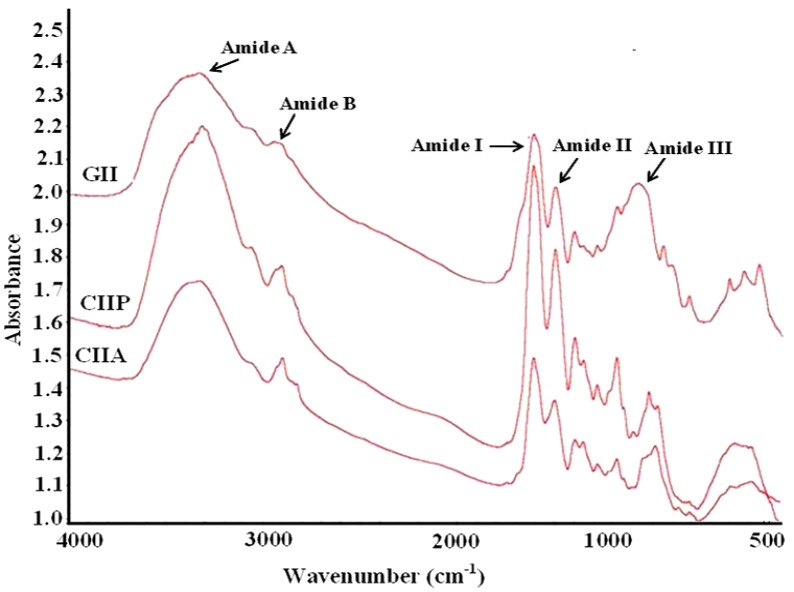
FTIR spectra of type II collagens and type II gelatin. CIIP: type II pepsin soluble collagen, CIIA: type II acid soluble collagen, GII: type II gelatin.

FTIR spectroscopy was employed to monitor the functional groups and secondary structure of type II collagens and type II gelatin. The frequencies at which major peaks occurred for type II collagens and gelatin are given in Table 3. The amide-A peak of CIIA, CIIP and GII appeared at 3340.38, 3331.13 and 3350.73 cm^−1^, respectively (Figure 6). This peak is generally associated with N–H stretching coupled with the hydrogen bond of a carbonyl group in a peptide chain.

The amide-B peak was observed at 2927.42, 2932.28 and 2948.13 cm^−1^ for CIIA, CIIP and GII, respectively. This peak represents the asymmetric stretching vibration of alkenyl C–H, as well as NH_3_^+^. The amide-A and amide-B peaks of type II gelatin appeared at higher wavenumbers than that of type II collagens. The higher wavenumbers of GII are likely due to the increased protein-protein intermolecular cross-linkages through hydrogen bonds of low molecular weight peptides, rather than high molecular weight collagens [19].

**Table 3:** General peak assignments of the FTIR spectra of type II collagens and type II gelatin isolated from shark cartilage. CIIP: type II pepsin soluble collagen, CIIA: type II acid soluble collagen, GII: type II gelatin.

Peak Wavenumber (cm^−1^)			
CIIP	CIIA	GII	Assignment
3331.13	3340.38	3350.73	Amide A: NH stretch coupled with a hydrogen bond
2932.28	2927.42	2948.13	Amide B: CH_2_ asymmetrical stretch
2882.11	2855.34	-	CH_3_-symmetric stretch: mainly proteins
1796.47	1734.76	-	Carbonyl C=O stretch: lipids
1659.84	1659.77	1657.77	Amide I: C=O stretch/hydrogen bond coupled with COO–
1550.67	1554.23	1551.26	Amide II: NH bend coupled with an CN stretch
1452.09	1452.16	1453.79	CH_2_ bend
1338.83	1338.22	1338.47	CH_2_ wag of proline
1239.69	1240.40	1237.81	Amide III: NH bend coupled with an CN stretch
1157.44	1161.1	1129.96	CO-O-C asymmetric stretch: glycogen and nucleic acids
1079.18	1079.52	-	C–O stretch
876.16	874.26	874.30	Skeletal stretch
616.96	611.58	597.11	Skeletal stretch

The amide-I peak was observed at 1659.77, 1659.84 and 1657.77 cm^−1^ for CIIA, CIIP and GII, respectively, which was similar to that of other fish collagens [19,27,31]. The amide-I region is mainly used for the analysis of the secondary structure of proteins [19]. The amide-I vibration mode is primarily due to the C=O stretching vibration of the peptide linkages (approximately 80%). The shift of the amide-I peak to a lower wavenumber is associated with the coiled structure of gelatin resulting from the heat denaturation during the isolation process [29].

The characteristic peak of the amide-II region of CIIA, CIIP and GII was observed at 1554.23, 1550.67 and 1551.26 cm^−1^, respectively. The amide-II vibration modes are attributed to the N–H in-plane bend (40%–60%) and the C–N stretching vibration (18%–40%). Muyonga *et al.* [19] also observed the amide II peak at 1540–1558 cm^−1^ for Nile perch skin collagen. The intensity of the amide peak is associated with the triple helical structure of collagen. In the present study, the intensity of the amide I peak of GII was lower than type II collagens and this indicated the loss of the triple helix structure of collagen during the isolation process. The protein structure of gelatin also confirmed the above statement (Figure 1). Therefore, FTIR spectra clearly indicated that the triple helix structure, molecular order and intermolecular cross-linkages of collagen varied from gelatin.

### 2.10. CD Spectra

The triple helix of collagen consisted of three molecular strands. The prolines are arranged in a left handed polyproline-II-helical conformation, and these helices coil together to form a right handed super helix. Circular dichroism spectroscopy (CD) is used to investigate the molecular order of collagen.

**Figure 7:**
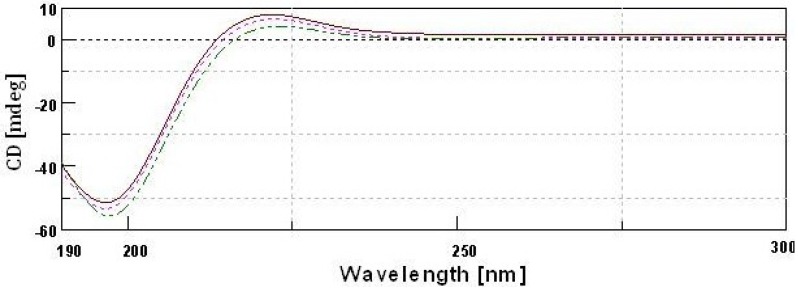
Circular dichroism spectra of type II collagens and type II gelatin. 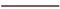 type II pepsin soluble collagen; 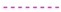 type II acid soluble collagen; 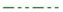 GII-type II gelatin.

CD spectra of type II collagens and type II gelatin exhibited a rotatory maximum at 221–221.5 nm, a minimum at 196–197 nm and a crossover point at 213.5–215.5 nm (Figure 7), which is characteristic of triple helical protein conformation [33]. The maximum and minimum rotatory CD spectra reported for eel skin collagen were 230 and 204 nm, respectively [26], and those of Adult black drum and sheepshead seabream bone were 220–221 and 197–199 nm, respectively [2]. However, for gelatin that was isolated by heat treatment, the CD spectra was similar to that of collagen. Aewsiri *et al.* [34] reported that after the complete denaturation of collagen, the characteristic triple helical positive peak at 220–230 nm disappeared and only a negative peak at 200 nm remained for gelatin. The triple helical structure of collagen can transform into a random coil when it is heated above its denaturation temperature [19]. The present study showed that the triple helical structures of type II gelatin were not completely destroyed during the isolation process. This is in agreement with the CD spectra of gelatin isolated from Amur sturgeon skin [35].

### 2.11. Antioxidant Activity

In the present study, the protective abilities of type II collagens and type II gelatin against oxidation was examined with the DPPH radical scavenging capacity and the reducing power. Natural or artificial antioxidants scavenge the DPPH radicals by donating a proton to the system. Type II collagens and type II gelatin were capable of scavenging hydroxyl radicals. The DPPH radical scavenging rate is 24%–16% for type II collagens and type II gelatin, which is lower than that of BHT (65%) (Table 2). CIIP exhibited higher scavenging activity than CIIA and GII. Zhu *et al.* [13] reported that the DPPH radical scavenging activity of PSC from sea cucumber was 45.58%, which was higher than our findings. It is thought that collagen can inactivate reactive oxygen species, reduce hydroperoxides, enzymatically eliminate specific oxidants, chelate pro-oxidative transition metals and scavenge free radicals, which may contribute to their antioxidant activities [13]. Moreover, certain specific amino acid residues and their sequences are thought to be responsible for antioxidant activities [15]. Zhong *et al.* [12] suggested that the antioxidant activities of the body wall of sea cucumber may be attributed to collagens. Zhuang *et al.* [14] reported that collagen from jellyfish exhibited improved antioxidant activity in animal mice skin by protecting the endogenous antioxidant enzymes and suggested that the high contents of glycine, proline, and hydrophobic amino acids of the collagen were responsible for the activity. In the present study, higher content of proline and hydrophobic amino acids (valine, leucine, isoleucine, cysteine and methionine) in CIIP than that of CIIA and GII also supports the superior antioxidant activity of CIIP.

The reducing power is determined by the ability of collagen to reduce ferric into ferrous ions. Similar to the DDPH activity, the reducing power was higher in CIIP than in CIIA and GII, with a value of 0.24 at 700 nm (Table 2). The present result was comparable to the porcine protein reducing power of 0.25 [36] and lower than that of the buckwheat protein (0.77–1.24) [37]. The reducing power of BHT was higher than the isolated collagen and gelatin. Li *et al.* [6] reported that the reducing ability increased with increasing concentrations of ASC from 1 to 5 mg/mL. The antioxidant property of proteins is primarily influenced by their size, configuration and amino acid composition [6]. Antioxidant activity of type II collagens and gelatin is one of the important properties for its use as a drug, especially for the treatment of RA. Some researchers have reported the effective inhibitory activity of cartilage type II collagen on rheumatoid arthritis [1,7,22]. There is substantial evidence that the CII-deficiency in transgenic mice results in embryos that die at birth and have skeletal malformations, thus indicating the importance of this protein in bone development [38]. Therefore, further investigations are required to identify specific antioxidant peptides from silvertip shark cartilage collagen and gelatin.

### 2.12. Microscopic Structure

**Figure 8:**
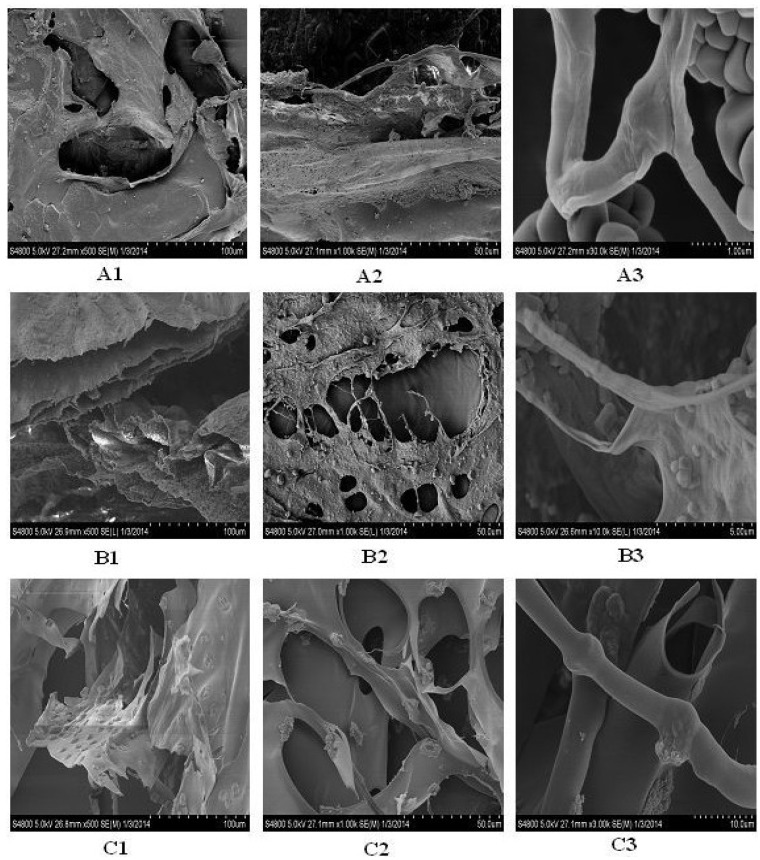
Scanning electron microscopic structure of type II collagens and type II gelatin isolated from silvertip shark cartilage. A1, A2, A3: type II pepsin soluble collagen; B1, B2, B3: type II acid soluble collagen; C1, C2, C3: type II gelatin.

The SEM microscopic structure showed a homogenous, porous, fibrillary and multi-layered structure of collagen and gelatin (Figure 8). The microstructure of CIIA and CIIP was a rough, fibril-dominated network with a coarse structural integrity. Conversely, the surface of gelatin was a smooth, less fibril-dominated network and was partially degraded. This similar structure was reported for collagen isolated from Amur sturgeon skin [31]. The microstructural changes in the collagen and gelatin are related to the functional properties. At a higher magnification, the collagen and gelatin had a fiber-like structure with an average size of 0.4 to 0.7 μm for CIIP and 0.75 to 1.5 μm for CIIA. An inter-linkage between CIIP and glycoprotein was observed at higher magnification. This structure was similar to the eel skin ASC and PSC structures reported by Veeruraj *et al.* [26]. Accordingly, the surface morphology of collagen greatly differs from that of gelatin due to isolation process.

The microstructural changes in collagen are related to the functional properties. The present SEM image showed that the shark collagens and gelatin had a cross-section with an inter-connected network pore configuration. Joseph *et al.* [39] suggested that the moderate pore size of collagen was suitable for *in vivo* studies and that the pore size of collagen was influenced by the water content during preparation. Earlier, Jansson *et al.* [40] reported that the collagen extracted from the cartilage of horse mackerel and croaker could be used as a biofilm or scaffold for wound healing purposes. In addition, other architectural features, such as pore shape, pore wall morphology and interconnectivity of collagen, have also been suggested for use in cell seeding, growth, gene expression, migration, mass transport, and new tissue formation. Generally, the uniform and regular network structure of collagen as a drug carrier is propitious for a well-proportioned drug distribution [41]. The microscopic structure of type II collagens and gelatin isolated from shark cartilage may provide a suitable biomaterial for a drug carrier system.

## 3. Experimental Section

### 3.1. Raw Materials

The cartilage (cartilaginous skeleton) of the silvertip shark was used as the raw material for the isolation of fish collagen and gelatin and was obtained from a private fish processing plant, M/s. Yueqing Ocean Biological Health Care Product Co., Ltd. Zhejiang province, Shanghai, China. The cartilage was washed with potable water and cut into small pieces prior to the isolation of collagen and gelatin. Moisture, ash, fat and protein content of the silvertip shark cartilage was determined according to AOAC [42] methods: No. 950.46, 928.08, 960.39 and 920.153, respectively. The sample hydroxyproline content was determined according to the method of Bergman and Loxley [43].

### 3.2. Isolation of Collagen and Gelatin

#### 3.2.1. Pretreatment of Shark Cartilage

The cartilage was homogenized in a tissue homogenizer using phosphate buffer (pH 6.5; 0.2 mol/L sodium dihydrogen phosphate and 0.2 mol/L disodium hydrogen phosphate heptahydrate). The entire process of collagen isolation was performed at 4 °C. The homogenate was treated with double-distilled water at a ratio of 1:6 (*w/v*) for 24 h to remove water soluble substances. The cartilage was decalcified with 0.5 Methylenediaminetetraacetic acid (EDTA) (pH 7.4) at a ratio of 1:10 (*w/v*) for 48 h. The solution was replaced every 12 h. This pretreated shark cartilage was used for the further isolation of acid soluble collagen (ASC) and pepsin soluble collagen (PSC).

#### 3.2.2. Isolation of ASC and PSC

Type II collagen (CII) was isolated from the shark cartilage according to the method of Kittiphattanabawon *et al.* [21] with modification. The pretreated shark cartilage was soaked into 0.5 M acetic acid (1:6, *w/v*) for 4 days with continuous shaking and the extracts were centrifuged at 10,000 rpm for 30 min at 4 °C. The supernatant was collected and salted out by adding 2 M NaCl. The precipitates were re-dissolved in a minimum volume of 0.5 M acetic acid and dialyzed against distilled water for 2 days until a neutral pH was obtained. The dialyzed sample was lyophilized (Labconco Freezone 2.5, Kansas City, MI, USA) and further referred to as ASC. For the isolation of PSC, the pretreated shark cartilage was soaked in 0.5 M acetic acid containing 1% pepsin (1:6, *w/v*) and then followed the above procedure.

#### 3.2.3. Isolation of Gelatin

Type II gelatin (GII) was isolated according to Jeevithan *et al.* [4]. Briefly, the shark cartilage was treated twice with 0.2% NaOH at a ratio of 1:6 *w/v* for 45 min to remove the non-collagenous protein. After thorough washing, cartilage was treated twice with 0.2% H_2_SO_4_ at a ratio of 1:6 *w/v* for 45 min. Samples were then treated with 1% citric acid twice at a ratio of 1:6 *w/v* for 45 min. The final isolation was carried out with distilled water at a ratio of 1:1 *w/v* at 45°C for 24 h. The extract was filtered through Whatman No. 4 filter paper under vacuum, lyophilized and used for further characterization.

### 3.3. Purification by Gel Filtration Chromatography

Gel filtration chromatography was performed to purify CIIA, CIIP and GII. In brief, samples (100 mg) were dissolved in 10 mL of a running buffer (20 mM sodium acetate buffer, pH 4.8) and were applied to a Sephadex G-100 (Sigma, Shanghai, China) column (25.0 × 3.0 cm). The column was previously equilibrated with the same buffer until A_230_ was less than 0.05 to achieve baseline correction. The elution volume was 200 mL, and the flow rate was 1 mL/min. The protein fractions were identified by UV absorbance spectroscopy and 5 mL fractions were collected. As shown in Figure 1b, the collagen peak was pooled and salted out by addition of 2 M NaCl. The precipitate was dialyzed against distilled water and freeze dried.

### 3.4. Sodium Dodecyl Sulfate-Polyacrylamide Gel Electrophoresis (SDS-PAGE)

The protein pattern was analyzed using sodium dodecyl sulfate poly-acrylamide gel electrophoresis (SDS-PAGE) according to the method of Laemmli [44] with modification. Briefly, the purified samples were dissolved in 5% SDS, kept in a water bath at 60 °C for 20 min and centrifuged at 3500 rpm. The supernatant was mixed with a sample buffer (1:1) containing Tris HCl (pH 6.8; 12.1 g Tris base was dissolved in 80 mL distilled water and the pH was adjusted with concentrated HCl), 1% 2-mercaptoethanol, 40% sucrose, 20% glycerol, 0.02% bromophenol blue and 1% SDS. The mixtures were loaded onto a polyacrylamide gel, composed of 7.5% separating gel and 4% stacking gel, and were subjected to electrophoresis at a constant current of 50 mA. After electrophoresis, gels were fixed with a mixture of 5:1 methanol:acetic acid would be composed of 83.3% and 16.7% methanol and acetic acid, respectively for 1 h. This was followed by staining with 0.5% Coomassie blue R-250 in 150% methanol and 50% acetic acid for 30 min. Finally, the gels were destained with a mixture of 300% methanol and 100% acetic acid for 2 h.

### 3.5. Peptide Mapping

Peptide mapping was performed according to the method of Kittiphattanabawon *et al.* [28] with modification. Samples (5 mg) were dissolved in 1 mL of 0.1 M Tris-HCL (pH 6.8) buffer containing 0.5% SDS and heated at 100 °C for 2 h. To initiate digestion, 20 μL of the enzyme solution, V8-protease (EC 3.4.21.19, Sigma-Aldrich, Shanghai, China), with a concentration of 5 μg/mL was added to each mixture. The reaction mixtures were then incubated at 37 °C for 1 h and the proteolysis was halted by increasing the temperature to 100 °C for 5 min. Peptides generated by the protease digestion were separated by SDS-PAGE using 10% separating gel and 4% stacking gel as previously described.

### 3.6. Viscosity

Samples (0.03%) were dissolved in 0.1 M acetic acid and the viscosity was measured using a Brookfield LVDV-II+P viscometer (Brookfield Engineering Laboratories Ltd., Middleboro, MA, USA) equipped with an ultra-low viscosity adapter at a speed of 90 rpm. Samples were heated using a rotary water bath from 5 to 40 °C. The sample solutions (20 mL) were incubated for 30 min at each temperature prior to measurement. The relative viscosity was calculated compared with that obtained at 5 °C.

### 3.7. Collagen Solubility Test

Optimum solubility at different pH and salt concentrations was determined according to the method of Jongjareonrak *et al.* [24]. Collagen samples were dissolved in 0.5 M acetic acid with gentle stirring at 4 °C for 12 h to obtain a final concentration of 6 mg/mL.

#### 3.7.1. Effect of pH

The collagen solution (5 mL) was transferred into a series of centrifuge tubes, adjusted to pH values ranging from 1 to 10 by addition of the appropriate amount of 6 M NaOH or 6 M HCl. The resulting sample solution totaled 10 mL with distilled water. The solution was stirred gently for 30 min at 4 °C and centrifuged at 5000× *g* for 30 min. An aliquot (1 mL) of the supernatant was collected from each tube and the protein content was measured by the Lowry method [45]. The relative solubility of collagen was calculated compared with the pH rendering the highest solubility.

#### 3.7.2. Effect of NaCl

The collagen solution (5 mL) was mixed with 5 mL of cold NaCl in acetic acid of various concentrations (0%–12%, *w/v*) to obtain final concentrations of 1%–6% (*w/v*). The mixture was stirred gently at 4 °C for 30 min and centrifuged at 10,000× *g* for 30 min at 4 °C. The relative solubility was calculated compared with that of the salt concentration exhibiting the highest solubility.

### 3.8. UV Absorption Spectrum

UV absorption spectrum of type II collagen samples was measured using a UV spectrophotometer. Samples were dissolved in 0.5 M acetic acid and UV spectra were measured between 190 and 400 nm at a scan speed of 2 nm/s with an interval of 1 nm.

### 3.9. Amino Acid Profiling

The collagen samples were hydrolyzed under reduced pressure in 6 M HCl at 110 °C for 24 h. Amino acid composition was analyzed using an amino acid analyzer (Hitachi L-8800, Tokyo, Japan). The amino acid content is expressed as the number of residues/1000 residues.

### 3.10. Fourier Transform Infrared Spectroscopy (FTIR)

FTIR spectra of the samples were obtained using a Nicolet 6700-Fourier transform infrared spectrometer (Thermofisher Scientific Inc., Waltham, MA, USA) equipped with a DLaTGS detector. The lyophilized samples (5 mg) were mixed with dried KBr (100 mg), ground in a mortar and pestle and subjected to a pressure of approximately 5 × 10^6^ Pa in an evacuated die to produce a 13 × 1 mm clear transparent disk. The absorption intensity of the peaks was calculated using the base-line method. The resultant spectra were analyzed using ORIGIN 8.0 software (Thermo Nicolet, Madison, WI, USA).

### 3.11. Circular Dichroism (CD)

The molecular conformations of collagen and gelatin were assessed by CD using a spectropolarimeter (Jasco J-810, Shanghai, China) according to the method of Cao *et al.* [22] with modification. Briefly, the samples were dissolved in 0.1 M acetic acid to obtain a final concentration of 0.1 mg/mL and were stirred for 6 h. Then, the sample solutions were placed in a quartz cell with a path length of 10 mm. The spectra were recorded between 190 and 300 nm at 25 °C. The acetic acid spectrum was used as a reference and CD spectra of the samples were obtained after subtracting the reference spectrum.

### 3.12. Scanning Electron Microscopy (SEM)

Morphological characteristics of the isolated collagens and gelatin were visualized by SEM-S4800 (Hitachi, Tokyo, Japan). Collagen samples were mounted on specimen stubs with two-sided carbon tape. The sample surface was sputter-coated with a thin layer (~8–10 nm) of gold ions using a sputter-coater. The samples were then introduced into the specimen chamber and examined for surface morphology at a 20 kV accelerating voltage.

### 3.13. Thermal Stability

Differential scanning calorimetry (DSC) was conducted following the method of Rochdi *et al.* [46]. The samples were rehydrated with deionized water at a solid/solution ratio of 1:10 (*w/v*). DSC was performed using a differential scanning calorimeter (Model DSC822e, Mettler-Toledo GmbH, Greifensee, Switzerland). The temperature calibration was conducted using an indium standard. Samples were weighed into aluminum pans and sealed. Subsequently, samples were scanned at 5 °C/min from 20 to 120 °C using ice water as the cooling medium. An empty pan was used as the reference. The denaturation and melting temperatures were estimated from the DSC thermogram.

### 3.14. Antioxidant Activity

#### 3.14.1. DPPH Radical Scavenging Assay

DPPH radical scavenging activity was acquired based on the method of Shimada *et al.* [47]. Briefly, the test sample (500 μL) was added to 500 μL of 99.5% ethanol and 125 μL of 99.5% ethanol containing 0.02% DPPH. The mixture was kept in the dark at room temperature for 60 min before measuring absorbance at 517 nm. In the blank, the sample was replaced with distilled water (500 μL) and the above procedure was followed. Butylated hydroxytoluenum (BHT) was used as a positive control. A lower absorbance indicated higher DPPH scavenging activity. Radical scavenging activity was calculated as follows:

Radical scavenging activity = [(blank absorbance-sample absorbance)/blank absorbance] × 100%.

#### 3.14.2. Reducing Power

Protein solutions (1 mg) were mixed with 2.5 mL of phosphate buffer (0.2 M, pH 6.6) and 2.5 mL of 1.0% potassium ferricyanide and the mixture was incubated at 50°C for 20 min. Trichloroacetic acid (2.5 mL of a 10% solution) was added, and the mixture was centrifuged. The supernatant (2.5 mL) was mixed with water (2.5 mL) and 0.1% ferric chloride (0.5 mL), and the absorbance was measured at 700 nm. Higher absorbance indicated higher reducing power. BHT was used as a reference antioxidant.

## 4. Conclusions

Collagen and gelatin was isolated from shark cartilage using an improved isolation method with a high denaturation temperature. Compared with type II gelatin, CIIP was richer in proline and alanine and lower in hydroxyproline. The homogenous, porous, fibrillary and multi-layered structure of type II collagens and type II gelatin are good characteristics of biomaterials used in cell attachment. In conclusion, the solubility, susceptibility to proteolytic enzymes, high denaturation temperature and efficient antioxidant activities suggest that silvertip shark cartilage type II collagens and type II gelatin have promising potential for use in various practical applications in biomedical industries.
